# Deletion of *Mea6* in Cerebellar Granule Cells Impairs Synaptic Development and Motor Performance

**DOI:** 10.3389/fcell.2020.627146

**Published:** 2021-02-25

**Authors:** Xin-Tai Wang, Lin Zhou, Xin-Yu Cai, Fang-Xiao Xu, Zhi-Heng Xu, Xiang-Yao Li, Ying Shen

**Affiliations:** ^1^Department of Physiology, School of Medicine, Zhejiang University, Hangzhou, China; ^2^Department of Psychiatry, Sir Run Run Shaw Hospital, School of Medicine, Zhejiang University, Hangzhou, China; ^3^State Key Laboratory of Molecular Developmental Biology, CAS Center for Excellence in Brain Science and Intelligence Technology, Institute of Genetics and Developmental Biology, Chinese Academy of Sciences, Beijing, China; ^4^Department of Neurobiology, School of Brain Science and Brain Medicine, Zhejiang University, Hangzhou, China

**Keywords:** Mea6, malformation, motor performance, vGluT1, granule cell, Fahr’s syndrome

## Abstract

The cerebellum is conceptualized as a processor of complex movements. Many diseases with gene-targeted mutations, including Fahr’s disease associated with the loss-of-function mutation of meningioma expressed antigen 6 (*Mea6*), exhibit cerebellar malformations, and abnormal motor behaviors. We previously reported that the defects in cerebellar development and motor performance of Nestin-Cre;*Mea6*^F/F^ mice are severer than those of Purkinje cell-targeted pCP2-Cre;*Mea6*^F/F^ mice, suggesting that Mea6 acts on other types of cerebellar cells. Hence, we investigated the function of Mea6 in cerebellar granule cells. We found that mutant mice with the specific deletion of *Mea6* in granule cells displayed abnormal posture, balance, and motor learning, as indicated in footprint, head inclination, balanced beam, and rotarod tests. We further showed that Math1-Cre;*Mea6*^F/F^ mice exhibited disrupted migration of granule cell progenitors and damaged parallel fiber-Purkinje cell synapses, which may be related to impaired intracellular transport of vesicular glutamate transporter 1 and brain-derived neurotrophic factor. The present findings extend our previous work and may help to better understand the pathogenesis of Fahr’s disease.

## Introduction

The cerebellum has been conceptualized as a processor of complex movements and is also endowed with essential roles in cognitive and emotional behaviors (Su et al., 2020). In essence, the cerebellar cortex can be trained to run routine operations that result in skillful movements triggered by high-level commands from the cerebral cortex. The structure of the cerebellum is extremely conserved among species from rodents to human, and its development involves the integration of intrinsic and extrinsic events controlled by multiple genetic cascades. Many gene-targeted mutations cause cerebellar malformations and impair motor and non-motor behaviors (Su et al., 2020). For example, the patients with Fahr’s disease (Lemos et al., 2011), a neurological inheritance disorder (Oliveira et al., 2007), exhibit unsteady gaits, and severe degeneration in brain regions controlling movements (Moskowitz et al., 1971; Geschwind et al., 1999; Saleem et al., 2013).

Meningioma expressed antigen 6 (Mea6), initially found in tumor tissues (Heckel et al., 1997; Comtesse et al., 2002; Kalniņa et al., 2008), is highly expressed in the central nervous system (CNS). Clinical evidence shows that a loss-of-function mutation of Mea6 might be associated with Fahr’s syndrome (Lemos et al., 2011). Utilizing conditional knockout of *Mea6* driven by Nestin-Cre, Zhang et al. (2018) show that Mea6 is critical to neural development and dendrite outgrowth in the cerebral cortex. However, this study provides limited insights on how Mea6 plays roles in CNS development because Nestin-driven Cre recombinase inevitably affects all types of neural cells in the CNS. Instead, we report distinct cerebellar development and motor performance between Nestin-Cre;*Mea6*^F/F^ and Purkinje cell-targeted pCP2-Cre;*Mea6*^F/F^ mice (Wang et al., 2019). While Nestin-Cre;*Mea6*^F/F^ mice have shrunken cerebellum and lobules, pCP2-Cre;*Mea6*^F/F^ mice merely display extensive self-crossings of Purkinje cell dendrites without changing cyto-architecture of the cerebellum (Wang et al., 2019). These results suggest that Mea6 influences the development of other types of cerebellar cells beyond Purkinje cells.

Cerebellar granule cell, being the most numerous cell type, arises from the rhombic lip and forms a dense and distinct layers of cerebellar cortex. At the early developmental stage, granule precursor cells proliferate and differentiate to granule cells, which further migrate from the external granular layer (EGL) inwards to the internal granular layer (IGL). Granule cells receive afferent information from mossy fibers and innervate with Purkinje cells *via* parallel fibers. It has been established by physiological experiments and computational theories that granule cells are the ground of cerebellar circuitry and motor memories. Here, we investigated the contribution of Mea6 in cerebellar development and motor functions by deleting *Mea6* specifically in granule cells. Our results showed that the deletion of *Mea6* in granule cells led to severe motor symptoms during the posture, balance, and motor learning tests.

## Materials and Methods

### Animals

All experiments were approved by the Animal Experimentation Ethics Committee of Zhejiang University. Mice were kept at the Experimental Animal Center of Zhejiang University under temperature-controlled condition on a 12:12 h light/dark cycle. *Mea6*^F/F^ mice described previously (Wang et al., 2019). Math1-Cre;*Mea6*^F/F^ mice were obtained by crossing *Mea6*^F/F^ mice with Math1-Cre mice, which were obtained from Dr. Wei Mo (Xiamen University, Xiamen, China) (Kim et al., 2014). The resulting offspring were genotyped using PCR of genomic DNA (*Mea6* floxP fragment, F: 5′-GAC ACT TGA CCC CTC CTC TCC-3′; R: 5′-AAC GGC TCA TGC TTG CTA ACC-3′; Math1-cre, F: 5′-TGC AAC GAG TGA TGA GGT TC-3′; R: 5′-GCT TGC ATG ATC TCC GGT AT-3′). All experiments were performed blind to genotypes in age-matched littermates of either sex.

### Antibodies and Reagents

Antibodies against GAPDH, GluA1, GluA2, NeuN, and synaptophysin were from Millipore (Billerica, MA, United States). Antibodies against Bip, Robo2, Sema6A, Synapsin-1, Munc18-1, and 5-bromo-2′-deoxyuridine (BrdU) were from Abcam (Cambridge, United Kingdom). Antibodies against γ-protocadherin (γ-pcdh), Rab3A, Rim1, and Munc13-1 were from Synaptic Systems (Gottingen, Germany). Antibody against Slit2 was from Proteintech (Rosemont, IL, United States). Antibody against TrkB was from Cell Signaling (Danvers, MA, United States). Anti-vesicular glutamate transporter 1 (vGluT1) antibody was a gift from Dr. Masahiko Watanabe (Hokkaido University, Sapporo, Japan). Antibodies against both Mea6 and calbindin were from Sigma-Aldrich (St. Louis, MO, United States). Antibodies to β-tubulin and brain-derived neurotrophic factor (BDNF) were from Santa Cruz Biotechnology (Dallas, TX, United States). Goat anti-mouse and anti-rabbit IgG horseradish peroxidase-conjugated were from Thermo Fisher (Waltham, MA, United States). DAPI and Alexa Fluor-conjugated secondary antibody was from Invitrogen (Carlsbad, CA, United States). Protease inhibitor cocktail was from Roche (Mannheim, Germany). Nissl was from Beyotime (Shanghai, China). Other chemicals were from Sigma unless stated otherwise.

### Purification of Endoplasmic Reticulum (ER)

Endoplasmic reticulum fractions were purified according to previous work (Hammond et al., 2012; Wang et al., 2019). A centrifugation (700 × *g*; 10 min) was used to remove nuclei and large cellular debris from homogenized cerebellar tissues. A subsequent 15,000 × *g* (10 min) of supernatant was performed to pellet mitochondria. The resulting supernatant was loaded onto a three-layered sucrose gradient and centrifuged at 126,000 × *g* for 70 min on an ultracentrifuge. The white band between the top and 1.3 M-sucrose layers was collected, which was gently mixed by inversion with ice cold MTE solution supplemented with protease inhibitors. This mixture was centrifuged at 126,000 × *g* for 45 min resulting in a large and translucent pellet.

### RT-PCR

The contents of individual granule cells (P21) were harvested as described in previous work (Zhou et al., 2017). The tip of a conventional patch-clamp pipette was placed tightly on the soma of a selected granule cell and a gentle suction was applied. After complete incorporation of the soma, the negative pressure was released and the pipette was quickly removed from the bath. The harvested contents were subjected to RT-PCR using OneStep Kit (Qiagen, Germany). Forward (F) and reverse (R) primers used for amplification were as follows: *Mea6*, F: 5′-GTT GAA GGA TCA CAA ATA TC-3′; R: 5′-TCC TTT TTG AAA TAT CAG CC-3′; *Math1*, F: 5′-GAG TGG GCT GAG GTA AAA GAG T-3′; R: 5′-GGT CGG TGC TAT CCA GGA G-3′; *Gapdh*, F: 5′-GGT GAA GGT CGG TGT GAA CG-3′; R: 5′-CTC GCT CCT GGA AGA TGG TG-3′.

### Western Blot

The protein concentration was determined using BCA protein assay. Equal quantities of proteins were loaded and fractionated on SDS-PAGE, transferred to PVDF membrane (Immobilon-P, Millipore), immunoblotted with antibodies, and visualized by enhanced chemiluminescence (Thermo). The dilutions of antibodies were MEA6 (1:4,000), Slit2 (1:1,000), Robo2 (1:1,000), β-tubulin (1:2,000), GAPDH (1:20,000), γ-pcdh (1:2,000), BDNF (1:1,000), TrkB (1:1,000), Bip (1:5,000), Sema6A (1:1,000), vGluT1 (1:2000), Rab3A (1:2000), synapsin-1 (1:10000), Rim1 (1:1000), Munc18-1 (1:40000), synaptophysin (1:40000), Munc13-1 (1:1000), GluA1 (1:2000), GluA2 (1:2000), and secondary antibodies (1:10,000). Film signals were digitally scanned and quantitated using ImageJ 1.42q (NIH).

### Immunohistochemistry

Frozen sagittal sections (30 μm) were cut and placed in blocking solution for 1 h at room temperature (RT). After washing with PBS, sections were incubated with primary antibodies overnight at 4°C and incubated with secondary antibodies for 3 h at RT. Primary antibody dilutions used for immunohistochemistry were calbindin (1:500), NeuN (1:500), BrdU (1:400), and Mea6 (1:250). Alexa Fluor 488-conjugated goat anti rat IgG, Alexa Fluor 488-conjugated goat anti mouse IgG, Alexa Fluor 594-conjugated goat anti-mouse IgG antibody, Alexa Fluor 488-conjugated goat anti-rabbit IgG antibody and Alexa Fluor 594-conjugated goat anti-rabbit IgG antibody were diluted at 1:1,000. All antibodies were diluted in PBS containing 1% BSA and 1% normal goat serum.

### BrdU Labeling

Mice (P7) were injected intraperitoneally with 50 mg/kg BrdU and sacrificed for labeling observation after 5 days. After denaturing DNA (2 N HCl; 40°C; 30 min), brain sections (30 μm) were washed with sodium tetraborate (0.1 M). Sections were immunostained using BrdU and NeuN antibody.

### Nissl Staining

Nissl staining was performed using Nissl staining Kit (Beyotime). Sagittal cerebellar slices (30 μm) were immersed in Nissl staining solution for 5 min, rinsed with distilled water, dehydrated in ethanol, and cleared in xylene. Images of cerebellar cortex were captured using a light microscope.

### Transmission Electron Microscopy (TEM)

Mouse brains were removed and stored at 4°C for 2.5 h in fixative. Sagittal slices (300 μm) were prepared and rectangular molecular layer of lobules IV–V were separated. The slices were then rinsed six times with 0.1 M PB and post-fixed in 1% OsO_4_ for 30 min. Slices were then rinsed three times with ddH_2_O and stained with 2% uranyl acetate for 30 min at RT. After dehydrating, the samples were embedded in an epoxy resin. Ultrathin sections (90 nm) were cut using an ultra-microtome (Leica), stained with lead citrate solution, and mounted on grids. EM images were captured at 11,000× and 68,000× magnification using a TEM (FEI, Hillsboro, OR, United States). Parallel fiber-Purkinje cell synapses were identified by asymmetrical and short contacts, which were distinct from GABAergic or climbing fiber synapses (Ichikawa et al., 2016). ImageJ was used to count the numbers of synapse and vesicles per bouton.

### Slice Preparation

Sagittal sections of cerebellar vermis (300 μm) were prepared from anesthetic mice (P20–21) using a vibrating tissue slicer (Leica VT1000S) and ice-cold standard artificial cerebrospinal fluid (aCSF) containing (in mM): 125 NaCl, 2.5 KCl, 1.25 NaH_2_PO_4_, 1 MgCl_2_, 2 CaCl_2_, 26 NaHCO_3_, and 25 D-glucose, bubbled with 95% O_2_/5% CO_2_. After recovery for 30 min at 37°C, slices were placed in a submerged chamber that was perfused at 2 ml/min with aCSF. Patch clamp electrodes (3–5 MΩ) were filled with an intracellular solution composed of (in mM) 134 K-gluconate, 6 KCl, 4 NaCl, 10 HEPES, 0.2 EGTA, 4 Na_2_ATP, 0.3 Na_3_GTP, and 14 Na_2_phosphocreatine (pH 7.3, OSM 290).

### Whole-Cell Recording

Purkinje cells were visualized under an upright microscope (BX51, Olympus) with a 40× water-immersion objective and equipped with infrared differential interference contrast enhancement. Whole-cell recordings were obtained with an Axon MultiClamp 700B amplifier (Molecular Devices). Currents were digitized at 10 kHz and filtered at 3 kHz. Miniature excitatory synaptic currents (mEPSCs) was recorded in whole-cell configuration in the presence of tetrodotoxin (TTX, 0.5 μM) plus Gabazine (10 μM). To obtain parallel fiber-EPSCs, standard patch pipettes were filled with aCSF and placed in the middle third of the molecular layer. EPSCs were induced by above-threshold parallel fiber stimulation delivered in the presence Gabazine (10 μM). Paired-pulse facilitation (PPF) was examined by paired stimulations at different intervals. Offline analysis was conducted using a sliding template algorithm (ClampFit 10, Molecular Device).

### Rotarod Test

Rotarod test was performed as previously described (Zhou et al., 2017). After the habituation to rotarod, mice (2 month) were tested twice a day at a time interval of 8 h for four consecutive days. In each session, the velocity of rotation increased at a constant acceleration of 9 rpm/min starting from 5 rpm.

### Elevated Beam Test

This test was performed according to previous work (Hartmann et al., 2014; Zhou et al., 2017). The movement of mice on a round plastic beam (length 50 cm and diameter 1 cm) 40 cm above a surface with bedding was recorded and analyzed. The percentage of steps with hindpaw slips during runs on the beam was calculated.

### Foot Print Test

To evaluate mice’s walking gait we used footprint test according to previous work (Zhou et al., 2017; Wang et al., 2019). Mice hindpaws were painted with non-toxic ink, and they were allowed to freely traverse a clear plexiglass tunnel (100 cm × 10 cm × 10 cm), with a sheet of white absorbent paper (100 cm × 10 cm) placed at the bottom of the track and a darkened cage at the end of the tunnel to encourage the mouse to run toward a dark and safe environment. The resulting tracks provided the spatial relationship of consecutive footfalls, from which the stride length and stance width were measured. Measurements for three-step cycles were averaged, considering a cycle as the distance from one pair of hind prints to the next. Footprints at the start and the end of the tunnel were excluded from the analysis as they corresponded to the initiation and termination of the movement.

### Measurement of Head Inclination

Mice were allowed to freely traverse a white plexiglass tunnel (100 cm × 10 cm × 10 cm) in front of a camera. The angle formed by the connection line of two eyes and horizontal plane was measured by Adobe Photoshop CS3.

### Statistical Analysis

Data were analyzed using GraphPad Prism 6.0 (GraphPad Software, San Diego, CA, United States), Excel 2003 (Microsoft, Seattle, WA, United States), and Igor Pro 6.0 (Wavemetrics, Lake Oswego, OR, United States). Data analysts were blind to experimental conditions until the data were integrated. Standard deviations for controls were calculated from the average of all control data. Statistical difference was determined using two-sided unpaired Student’s *t* test. The accepted level of significance was *p* < 0.05. *n* represents the number of preparations or cells. Data are presented as mean ± SEM.

## Results

### *Mea6* Was Specifically Deleted in Cerebellar Granule Cells in Math1-Cre;*Mea6*^F/F^ Mice

**FIGURE 1:**
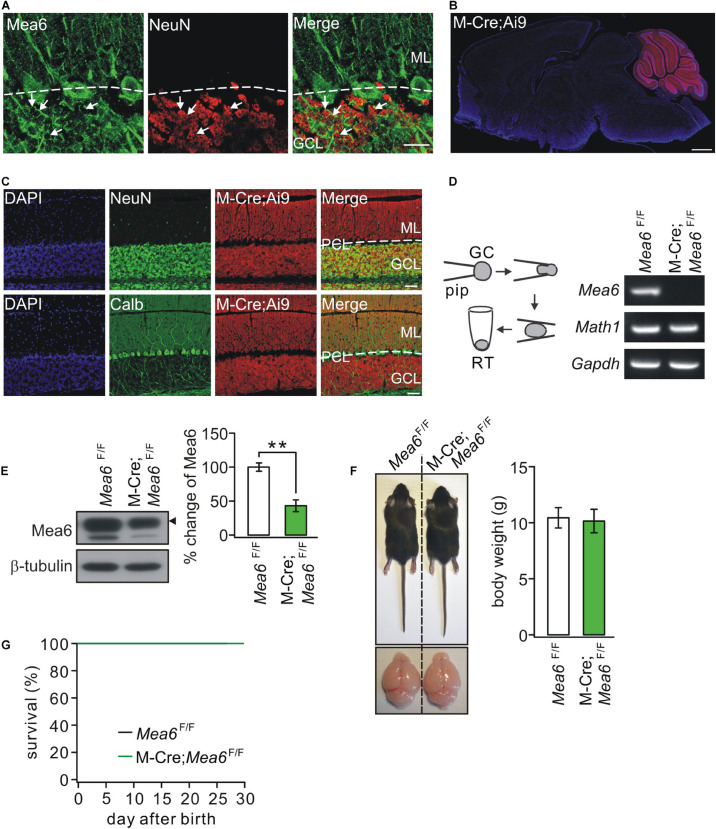
The ablation of *Mea6* in Math1(M)-Cre;*Mea6*^F/F^ mice. **(A)** The immunostaining of Mea6 (green) and NeuN (red) in mouse cerebellum. Arrows show Mea6-expressing granule cells. Scale bars: 20 μm. ML, molecular layer; GCL, granule cell layer. **(B)** Native tdTomato fluorescence in the whole brain from a Math1(M)-Cre;Ai9 mouse, indicating that Cre-recombinase is selectively expressed in the cerebellum. Scale bars: 1 mm. **(C)** Staining of NeuN or calbindin (Calb) with DAPI in the cerebellum of M-Cre;Ai9 mouse. Scale bars: 50 μm. **(D)** Granule cell contents of *Mea6*^F/F^ and Math1-Cre;*Mea6*^F/F^ mice (P21) were harvested using glass micropipettes (pip, OD 2 mm) and placed in a centrifuge tube. The contents collected from 10 cells were subjected to RT-PCR. A typical electrophoresis of *Mea6* (157 bp), *Math1* (151 bp), and *Gapdh* (233 bp) is show in the right (*n* = 5 trials). **(E)** Western blots of Mea6 in the cerebellum of *Mea6*^F/F^ and Math1-Cre;*Mea6*^F/F^ mice (P21), as indicated by the black triangle. The percentage changes of Mea6 were 100 ± 6% (*Mea6*^F/F^; *n* = 10) and 44 ± 9% (Math1-Cre;*Mea6*^F/F^; *n* = 10), *p* = 0.001 (unpaired *t* test). **(F)** The pictures of bodies and brains of *Mea6*^F/F^ and Math1-Cre;*Mea6*^F/F^ at P21. Average body weights were 10.4 ± 0.8 g (*Mea6*^F/F^; *n* = 10) and 10.2 ± 1.0 g (Math1-Cre;*Mea6*^F/F^; *n* = 10), *p* = 0.78 (unpaired *t* test). **(G)** Kaplan-Meier survival curves of *Mea6*^F/F^ (*n* = 59 mice) and Math1-Cre;*Mea6*^F/F^ mice (*n* = 59 mice). **p < 0.01.

Figure 1A shows that Mea6 was abundantly expressed in cell bodies and processes (parallel fibers) of cerebellar granule cells. Previous studies have shown that Mea6 is expressed in other brain regions besides the cerebellum (Zhang et al., 2018; Wang et al., 2019). Therefore, particular caution should be taken using Cre-loxp strategy to knock out *Mea6* in granule cells. We utilized the Math1-Cre mouse line (Kim et al., 2014), which targets to Math1+ neuronal precursors in developing rhombic lip that give rise to granule cells and unipolar brush cells (Englund et al., 2006; Schüller et al., 2008). To confirm the specificity, we crossed Math1-Cre and Ai9 lines and characterized the expression of Cre-recombinase by observing the tdTomato reporter in Math1-Cre;Ai9 mice. We found that tdTomato fluorescence was present merely in the cerebellum of these mice (Figure 1B), suggesting that the knockout mediated by Math1-recombinase is specific in the cerebellum. To examine whether Math1-recombination affects other cerebellar cells, we performed immunohistochemical staining using NeuN or calbindin antibodies and found that Math1-recombination was restricted to granule cell layer and parallel fibers (Figure 1C), suggesting that this recombination does not affect Purkinje cells and interneurons, which are located in Purkinje cell layer and molecular layer, respectively. Although Math1-recombination may affect unipolar brush cells as well, the influence should be marginal in our experiments because the number of these cells is very few compared to granule cells (Englund et al., 2006).

Next, we knock out *Mea6* gene in granule cells by mating *Mea6*^F/F^ mice with Math1-Cre transgenic mice. The knockout efficiency was confirmed by patch-clamp-based RT-PCR assay (Figure 1D). Western blots also showed that Mea6 expression was significantly reduced in Math1-Cre;*Mea6*^F/F^ mice (Figure 1E), which makes sense considering the great number of granule cells. We previously showed that both body weight and cerebellar size of Nestin-Cre;*Mea6*^F/F^ mice are reduced (Wang et al., 2019). Differently, these phenotypes did not differ between Math1-Cre;*Mea6*^F/F^ and *Mea6*^F/F^ mice (Figure 1F). In addition, the lifetime of Math1-Cre;*Mea6*^F/F^ mice was as long as that of *Mea6*^F/F^ mice (Figure 1G), similar to mutant mice with the specific deletion of *Mea6* in Purkinje cells (Pcp2-Cre;*Mea6*^F/F^) (Wang et al., 2019).

### *Mea6* Deletion in Granule Cells Impairs Motor Performance

**FIGURE 2:**
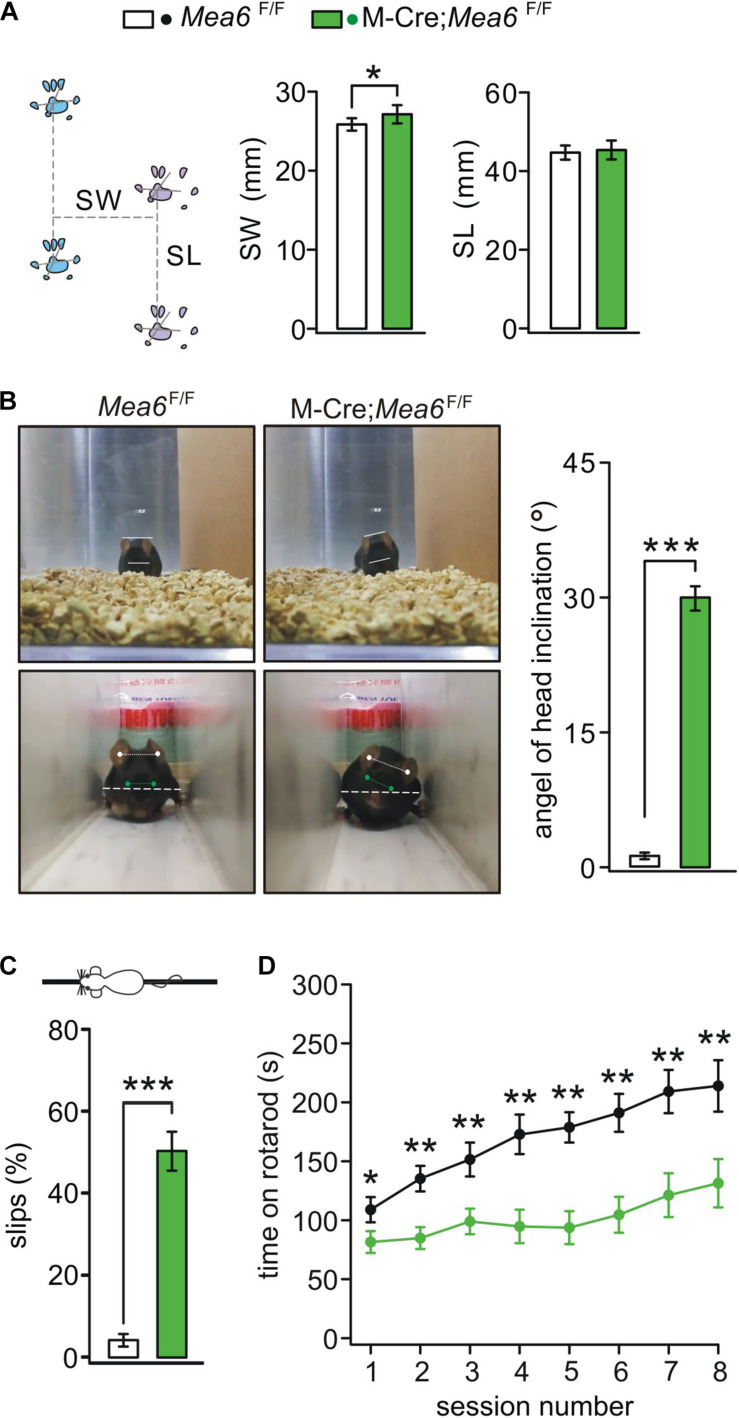
Abnormal gait and motor learning in Math1(M)-Cre;*Mea6*^F/F^ mice. **(A)** Footprints of *Mea6*^F/F^ and Math1-Cre;*Mea6*^F/F^ mice. Stride width (SW): 25.8 ± 0.8 mm (*Mea6*^F/F^; *n* = 16) and 27.1 ± 1.1 mm (Math1-Cre;*Mea6*^F/F^; *n* = 17), *p* = 0.02 (unpaired *t* test). Stance length (SL): 44.7 ± 1.8 mm (*Mea6*^F/F^; *n* = 16) and 45.3 ± 2.4 mm (Math1-Cre;*Mea6*^F/F^; *n* = 17), *p* = 0.52 (unpaired *t* test). **(B)** The upper panel shows a head inclination phenotype in a M-Cre;*Mea6*^F/F^ mouse during the free moving in the cage. The lower panel shows the measurement of head inclination when *Mea6*^F/F^ and M-Cre;*Mea6*^F/F^ mice traversed a white plexiglass tunnel (100 cm × 10 cm × 10 cm). The white lines show the alignments of ears and eyes. The average angels of head inclination: 1.2 ± 0.4° (*Mea6*^F/F^) and 30.0 ± 1.3° (Math1-Cre;*Mea6*^F/F^), *p* < 0.001 (unpaired *t* test). **(C)** The percentages of hindpaw slips during runs on an elevated horizontal beam. *Mea6*^F/F^: 4.1 ± 1.0% (*n* = 24). Math1-Cre;*Mea6*^F/F^: 48.4 ± 4.5% (*n* = 29), *p* < 0.001 (unpaired *t* test). **(D)** Time spent on the accelerating rotarod for *Mea6*^F/F^ (*n* = 13) and Math1-Cre;*Mea6*^F/F^ mice (*n* = 16) at P90. Session 1: 108.9 ± 10.6 s (*Mea6*^F/F^) and 81.5 ± 9.2 s (Math1-Cre;*Mea6*^F/F^), *p* = 0.04 (unpaired *t* test); Session 2: 135.3 ± 10.9 s (*Mea6*^F/F^) and 84.8 ± 9.3 s (Math1-Cre;*Mea6*^F/F^), *p* = 0.001 (unpaired *t* test); Session 3: 151.5 ± 14.3 s (*Mea6*^F/F^) and 98.9 ± 10.8 s (Math1-Cre;*Mea6*^F/F^), *p* = 0.004 (unpaired *t* test); Session 4: 187.5 ± 16.8 s (*Mea6*^F/F^) and 94.8 ± 14.2 s (Math1-Cre;*Mea6*^F/F^), *p* = 0.001 (unpaired *t* test); Session 5: 178.8 ± 12.8 s (*Mea6*^F/F^) and 93.7 ± 14.0 s (Math1-Cre;*Mea6*^F/F^), *p* = 0.001 (unpaired *t* test); Session 6: 191.1 ± 16.1 s (*Mea6*^F/F^) and 104.6 ± 15.2 s (Math1-Cre;*Mea6*^F/F^), *p* = 0.004 (unpaired *t* test); Session 7: 209.2 ± 18.3 s (*Mea6*^F/F^) and 121.25 ± 18.6 s (Math1-Cre;*Mea6*^F/F^), *p* = 0.002 (unpaired *t* test); Session 8: 213.9 ± 21.8 s (*Mea6*^F/F^) and 131.4 ± 20.5 s (Math1-Cre;*Mea6*^F/F^), *p* = 0.008 (unpaired *t* test). *p < 0.05, **p < 0.01, ***p < 0.001.

We previous reported that Nestin-Cre;*Mea6*^F/F^ mice displayed severe behavioral defects: they have abnormal limb-clasping reflex and foot prints, and perform poorly on elevated beam (Wang et al., 2019). In contrast, the behaviors of Pcp2-Cre;*Mea6*^F/F^ mice are almost normal except motor learning (Wang et al., 2019). These results suggest that *Mea6* deletion driven by Nestin-Cre may affect the development and/or functions of other types of cells except Purkinje cells. Accordingly, we examined whether the deletion of *Mea6* in granule cells causes motor deficits. Indeed, we found that Math1-Cre;*Mea6*^F/F^ mice walked abnormally with wider step stance of hindlimb without changing stride length (Figure 2A). Interestingly, Math1-Cre;*Mea6*^F/F^ mice (19 out of 19) displayed unexpected head inclination: their heads always tilted to either left or right when they walked freely in the cages, while *Mea6*^F/F^ mice did not (Figure 2B). To measure head tilt, mice were allowed to traverse a narrow plexiglass tunnel, where their free turning was limited. Through aligning ears and eyes, our results showed that average angels of head inclination were 1.2 ± 0.4° for *Mea6*^F/F^ mice (*n* = 16) and 30.3 ± 0.9° Math1-Cre;*Mea6*^F/F^ mice (*n* = 19). These experiments indicated abnormal walking gait of Math1-Cre;*Mea6*^F/F^ mice. We also found that Math1-Cre;*Mea6*^F/F^ mice were defective in other motor performances: They performed poorly when walking on an elevated beam with a higher number of hind-paw slips (Figure 2C) and spent much less time on rotating rod (Figure 2D). Taken together, our results indicated that Mea6 in granule cells is critical to motor behaviors.

### Disrupted Migration of Granule Cell Progenitors in Math1-Cre;*Mea6*^F/F^ Mice

**FIGURE 3:**
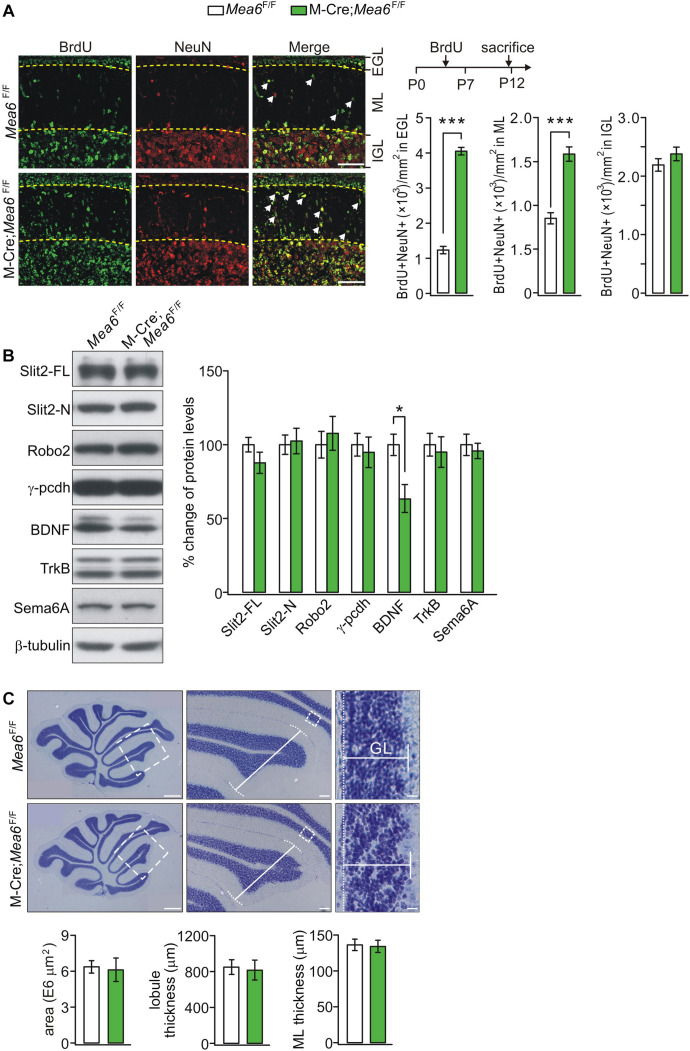
Deficiency in GCP migration in Math1(M)-Cre;*Mea6*^F/F^ mice. **(A)** Migrating GCPs in *Mea6*^F/F^ and Math1-Cre;*Mea6*^F/F^ cerebellum were treated with BrdU at P7 and labeled with anti-BrdU antibody after 5 days. White arrows show migrating GCPs in molecular layer (ML). Scale bars: 50 μm. The quantification of the numbers of NeuN+ and BrdU+ cells per 1 mm^2^ is shown in bar graphs. BrdU+/NeuN+ cells in EGL: 1220.4 ± 106.5 (*Mea6*^F/F^; *n* = 13) and 4061.7 ± 106.7 (Math1-Cre;*Mea6*^F/F^; *n* = 12), *p* < 0.001 (unpaired *t* test). BrdU+/NeuN+ cells in ML: 854.3 ± 64.5 (*Mea6*^F/F^; *n* = 13) and 1591.6 ± 81.8 (Math1-Cre;*Mea6*^F/F^; *n* = 12), *p* < 0.001 (unpaired *t* test). BrdU+/NeuN+ cells in IGL: 2199.4 ± 106.3 (*Mea6*^F/F^; *n* = 13) and 2388.8 ± 117.7 (Math1-Cre;*Mea6*^F/F^; *n* = 12), *p* = 0.24 (unpaired *t* test). **(B)** Protein levels of Slit2, Robo2, γ-pcdh, BDNF, TrkB, and Sema6A in the cerebellum of *Mea6*^F/F^ and Math1-Cre;*Mea6*^F/F^ mice at P20 (*n* = 6 pairs). β-tubulin was used as the control. BDNF: 100 ± 7% (*Mea6*^F/F^) and 63 ± 9% (Math1-Cre;*Mea6*^F/F^), *p* = 0.04 (unpaired *t* test). **(C)** Nissl staining of sagittal cerebellar sections from *Mea6*^F/F^ and Math1-Cre;*Mea6*^F/F^ mice at P25. The middle panel (Scale bars: 100 μm) is the higher magnification of left panel (Scale bars: 200 μm) and the right panel (Scale bars: 10 μm) is the higher magnification of middle panel, as indicated by white dashed boxes. Cerebellar area: 6.4 ± 0.5 E6 μm^2^ (*Mea6*^F/F^; *n* = 7) and 6.1 ± 1.0 E6 μm^2^ (Math1-Cre;*Mea6*^F/F^; *n* = 6), *p* = 0.13 (unpaired *t* test). Lobule III thickness: 849 ± 81 μm (*Mea6*^F/F^; *n* = 7) and 815 ± 109 μm (Math1-Cre;*Mea6*^F/F^; *n* = 7), *p* = 0.51 (unpaired *t* test). Thickness of granule cell layer (GCL; lobule III): 136 ± 8 μm (*Mea6*^F/F^; *n* = 7) and 134 ± 8 μm (Math1-Cre;*Mea6*^F/F^; *n* = 7), *p* = 0.43 (unpaired *t* test). *p < 0.05, ***p < 0.001.

The normal development of the cerebellum is essential to motor functions. The appearance of granule cell progenitors (G) in EGL and their migration to IGL are key features of cerebellar development (Alder et al., 1996). Having demonstrated motor deficits of Math1-Cre;*Mea6*^F/F^ mice, a question was whether the development of granule cells is disrupted. To answer this, the production and migration of GCPs in EGL of control and mutant mice at P7, a peak stage for postnatal GCP proliferation (Leffler et al., 2016), were examined using anti-NeuN antibody and BrdU antibody (Yang et al., 2019). Our results showed that the density of BrdU+ cells in IGL was similar between *Mea6*^F/F^ and Math1-Cre;*Mea6*^F/F^ mice at P12, 5 days after BrdU injection (Figure 3A). In contrast, the density of BrdU+ cells in molecular layer and EGL significantly increased in Math1-Cre;*Mea6*^F/F^ mice compared with *Mea6*^F/F^ mice at P12. These results implicated that the deletion of *Mea6* disrupts the migration of GCPs in Math1-Cre;*Mea6*^F/F^ mice.

The migration of GCPs is an intricate process that involves a number of secretary and cell-surface molecules, including cadherin (Horn et al., 2018), Slit2-Robo2 signaling (Xu et al., 2004; Guan et al., 2007; Geisen et al., 2008), BDNF-TrkB signaling (Borghesani et al., 2002; Wang et al., 2012), and semaphorin (Kerjan et al., 2005). Is the expression of these proteins changed by the deletion of *Mea6*? To answer this question, we measured the expression of Slit2, Robo2, γ-pcdh, BDNF, TrkB, and Sema6A. We found that the levels of Slit2, Robo2, γ-pcdh, TrkB, and Sema6A were not changed by specific deletion of *Mea6*, whereas the expression of BDNF significantly decreased in Math1-Cre;*Mea6*^F/F^ mice compared with *Mea6*^F/F^ mice (Figure 3B), implicating that the migration of GCPs is disrupted by BDNF down-regulation.

We continued to examine cerebellar cyto-architecture in adult mice. Using Nissl staining, we found that folia formation, lobular thickness, and GCL thickness of Math1-Cre;*Mea6*^F/F^ mice were not changed compared to *Mea6*^F/F^ mice (Figure 3C), indicating that cyto-architecture keeps intact in Math1-Cre;*Mea6*^F/F^ mice. From these results, we concluded that the deletion of *Mea6* in granule cells may delay the migration of GCPs, but not alter the structure of adult cerebellum. Hence, abnormal motor behaviors of Math1-Cre;*Mea6*^F/F^ mice are not due to changed cyto-architecture.

### Granule Cell Deletion of *Mea6* Damages Parallel Fiber-Purkinje Cell Synapse

**FIGURE 4:**
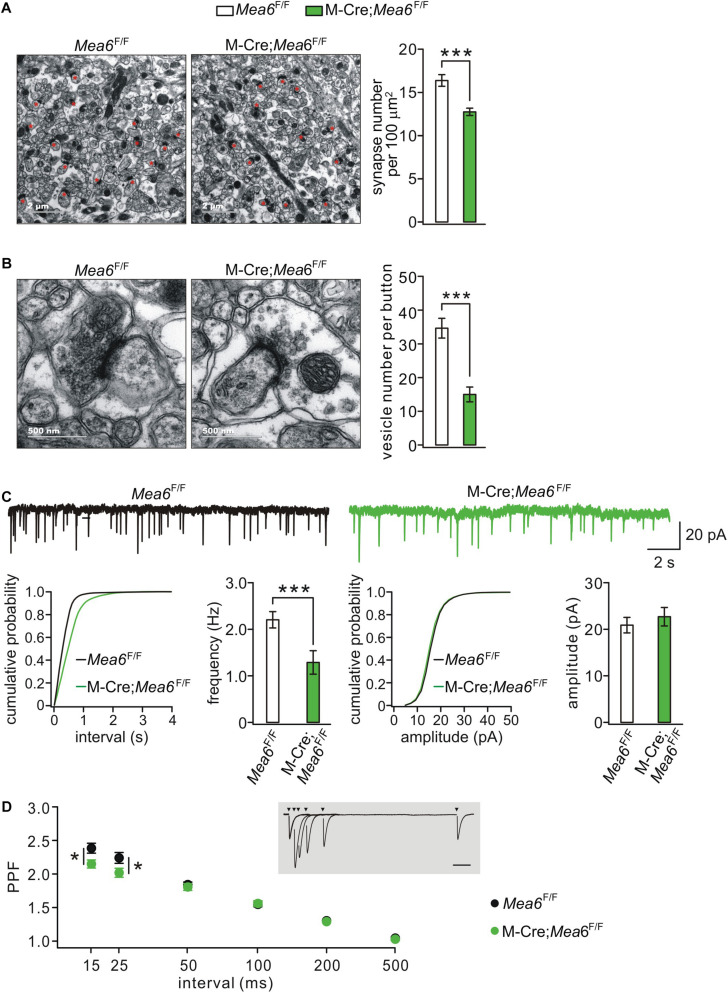
Granule cell-specific deletion of *Mea6* impairs synaptic formation and function. **(A)** Representative EM (11,000×) of parallel fiber-Purkinje cell synapses from *Mea6*^F/F^ and Math1(M)-Cre;*Mea6*^F/F^ mice (P30). Synapses comprising of parallel fiber boutons opposed to Purkinje cell spines are marked with red asterisks. Scale bars: 2 μm. Bar graphs show average numbers of synapses per 100 μm^2^ in *Mea6*^F/F^ (16.4 ± 0.7; *n* = 26) and Math1-Cre;*Mea6*^F/F^ mice (12.7 ± 0.4; *n* = 49), *p* < 0.001 (unpaired *t* test). **(B)** Representative EM (68,000×) of parallel fiber-Purkinje cell synapses from *Mea6*^F/F^ and Math1-Cre;*Mea6*^F/F^ mice (P30). Scale bars: 500 nm. Bar graphs show average numbers of total pre-synaptic vesicles per synapse in *Mea6*^F/F^ (34.7 ± 2.9; *n* = 26) and Math1-Cre;*Mea6*^F/F^ mice (15.0 ± 2.2; *n* = 27), *p* < 0.001 (unpaired *t* test). **(C)** Example Purkinje cell mEPSCs from *Mea6*^F/F^ and Math1-Cre;*Mea6*^F/F^ mice (P21–25). The lower panels show cumulative probabilities and statistics of frequency and amplitude of mEPSCs. Frequency: 2.3 ± 0.2 Hz (*Mea6*^F/F^; *n* = 15) and 1.6 ± 0.1 Hz (Math1-Cre;*Mea6*^F/F^; *n* = 19; *p* = 0.006), *p* = 0.006 (unpaired *t* test). Amplitude: 20.8 ± 0.9 pA (*Mea6*^F/F^; n = 15) and 23.7 ± 2.0 pA (Math1-Cre;*Mea6*^F/F^; *n* = 19), *p* = 0.34 (unpaired *t* test). **(D)** PPF as a function of interstimulus intervals in *Mea6*^F/F^ and Math1-Cre;*Mea6*^F/F^ mice (P21–23). The inset shows the superposition of PF-EPSCs evoked at different intervals in a WT cell. PPF ratios: 2.4 ± 0.1 (*Mea6*^F/F^; *n* = 12) and 2.1 ± 0.1 (Math1-Cre;*Mea6*^F/F^; *n* = 16) at 15 ms, *p* = 0.03 (unpaired *t* test); 2.2 ± 0.1 (*Mea6*^F/F^; *n* = 12) and 2.0 ± 0.1 (Math1-Cre;*Mea6*^F/F^; *n* = 16) at 25 ms, *p* = 0.03 (unpaired *t* test). *p < 0.05, ***p < 0.001.

Synaptogenesis and synaptic function between granule cells and Pukinje cells are also critical to the cerebellum-related motor behaviors (Su et al., 2020). We thereby used TEM to assess parallel fiber-Purkinje cell synapses identified by asymmetric synaptic contacts with Purkinje cell spines (Figure 4A; Peter et al., 2016). We found that the density of parallel fiber-Purkinje cell synapses was significantly reduced in Math1-Cre;*Mea6*^F/F^ mice (12.7 ± 0.4 synapses per 100 μm^2^; *n* = 49 slices of three mice) compared with *Mea6*^F/F^ mice at P21 (16.4 ± 0.7 synapses per 100 μm^2^; *n* = 26 slices of three mice; *p* < 0.001) (Figure 4A). Furthermore, the deletion of *Mea6* significantly decreased total number of vesicles at parallel fiber terminals (*Mea6*^F/F^: 34.7 ± 2.9 vesicles, *n* = 26 boutons; Math1-Cre;*Mea6*^F/F^: 15.0 ± 2.2 vesicles, *n* = 27 boutons; *p* < 0.001) (Figure 4B). These results suggest that the deletion of *Mea6* in granule cells impairs synaptic formation at parallel fiber-Purkinje cell synapses.

To further determine the effect of *Mea6* ablation on synaptic function, we recorded mEPSCs at parallel fiber-Purkinje cell synapses. Our results showed that mEPSC frequency was reduced in Math1-Cre;*Mea6*^F/F^ mice (1.6 ± 0.1 Hz; *n* = 19 cells) compared with *Mea6*^F/F^ mice (2.3 ± 0.2 Hz; *n* = 15 cells), whereas its mean amplitude did not differ between genotypes (*Mea6*^F/F^: 20.8 ± 0.9 pA, *n* = 15 cells; Math1-Cre;*Mea6*^F/F^: 23.7 ± 2.0 A, *n* = 19 cells) (Figure 4C). PPF of parallel fiber-Purkinje cell synapses was also examined using paired stimulations at different interstimulus intervals. Interleaved recordings indicated that average PPF recorded from Math1-Cre;*Mea6*^F/F^ mice changed with stimulation intervals: it was smaller than that from *Mea6*^F/F^ mice at intervals of <25 ms, but was unaltered at intervals larger than that (Figure 4D). Reduced PPF suggested a lower pre-synaptic release probability in Math1-Cre;*Mea6*^F/F^ mice, consistent with reduced synapses and pre-synaptic vesicles (Figure 4B). Overall, these data indicated that neurotransmitter release at parallel fiber-Purkinje cell synapses is impaired by *Mea6* deletion.

### Subcellular Transport of vGluT1 Is Interrupted in Math1-Cre;*Mea6*^F/F^ Mice

**FIGURE 5:**
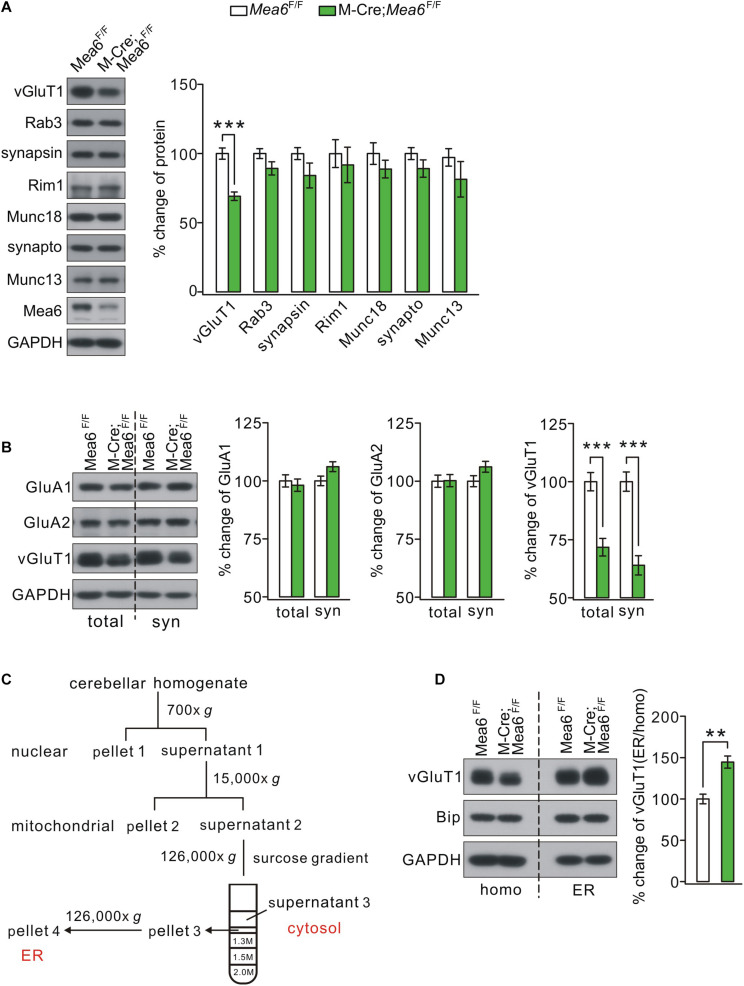
Mea6 deficiency affects the transport of vGluT1 from ER to Golgi apparatus. **(A)** Protein levels of vGluT1, Rab3A (Rab3), synapsin-1 (synapsin), Rim1, Munc18-1 (Munc18), synaptophysin (synapto), Munc13-1 (Munc13), and Mea6 in *Mea6*^F/F^ and Math1-Cre;*Mea6*^F/F^ cerebellum at P21. The results were obtained from eight pairs of mice. GAPDH was used as the loading control. **(B)** Protein levels of GluA1, GluA2, and vGluT1 in the cerebellum from *Mea6*^F/F^ and Math1-Cre;*Mea6*^F/F^ mice at P21. Six independent replicates were performed. GAPDH was used as the loading control. vGluT1 in total cerebellum: 100 ± 4% (*Mea6*^F/F^) and 72 ± 4% (Math1-Cre;*Mea6*^F/F^), *p* < 0.001 (unpaired *t* test). vGluT1 in cerebellar synaptosome: 100 ± 4% (*Mea6*^F/F^) and 64 ± 4% (Math1-Cre;*Mea6*^F/F^), *p* < 0.001 (unpaired *t* test). **(C)** A cartoon illustrating the procedures for the purification of subcellular organelles. More details are given in Experimental Procedures. The purification of ER was confirmed by the Western blotting assay of marker proteins. **(D)** Western blotting assay of vGluT1 in ER purified from *Mea6*^F/F^ and M-Cre;*Mea6*^F/F^ mouse cerebella. Bip was used as the internal control. vGluT1: 100 ± 6% (*Mea6*^F/F^) and 146 ± 7% (Math1-Cre;*Mea6*^F/F^). The experiment was performed eight times. *p* = 0.001 (unpaired *t* test). **p < 0.01, ***p < 0.001.

We continued to examine whether *Mea6* ablation affects the expression of pre-synaptic proteins that are involved in synaptic release and transmission. A number of pre-synaptic proteins related to synaptic function, including vGluT1, Rab3, synapsin-1, Rim1, Munc18-1, synaptophysin, and Munc13-1, were selected to mark potential pre-synaptic changes by *Mea6* ablation. Unexpectedly, we found that only vGluT1 significantly decreased in Math1-Cre;*Mea6*^F/F^ mice, while other pre-synaptic proteins were not changed (Figure 5A). The expression of vGluT1 was also detected at the synaptic level using different centrifugations (Zhou et al., 2015). Likewise, synaptic vGluT1 was significantly reduced in Math1-Cre;*Mea6*^F/F^ mice (Figure 5B). In contrast, the expression of α-amino-3-hydroxy-5-methyl-4-isoxazolepropionic acid receptor (AMPAR) subunits, including GluA1 and GluA2, was unchanged at synaptic level (Figure 5B).

Previous work showed that Mea6 participates in the trafficking of exogenous protein (Zhang et al., 2018) and endogenous Slit2 (Wang et al., 2019) between ER and Golgi apparatus. We next investigated whether the subcellular trafficking of vGluT1 is affected by *Mea6* deletion taking advantage of a series of centrifugations to purify organelles (Figure 5C; Wang et al., 2019). The purification of ER fraction was confirmed with distinct organelle markers, PDI (ER), γ-adaptin (Golgi apparatus), YY1 (nuclei), PSD95 (synapse), VDAC (mitochondria), and Rab11 (endosome) (Wang et al., 2019). Our results indicated that Bip, a marker molecule for ER (Haefliger et al., 2011; Shimizu et al., 2017; McLelland et al., 2018), was not changed in Math1-Cre;*Mea6*^F/F^ mice (Figure 5D), suggesting that ER is not affected by granule cell deletion of *Mea6*. Similar to Slit2 expression in pCP2-Cre;*Mea6*^F/F^ mice (Wang et al., 2019), the expression of vGluT1 significantly increased in ER fraction of Math1-Cre;*Mea6*^F/F^ mice compared to that in *Mea6*^F/F^ mice (Figure 5D), indicating that the deletion of *Mea6* tethers vGluT1 in ER and then decreases its expression.

## Discussion

We previously reported that Mea6 is essential to cerebellar development and motor performance (Wang et al., 2019): Nestin-Cre-induced knockout of *Mea6* leads to defective adult cerebellum and impaired motor performance; Purkinje cell-specific deletion of *Mea6* does not change lobular formation, but causes self-crossings of Purkinje cell dendrites and impairs motor learning. The present findings extended previous work by demonstrating the function of Mea6 in granule cells. We found that Math1-Cre;*Mea6*^F/F^ mice exhibited disrupted migration of GCPs, reduced synaptogenesis, and damaged parallel fiber-Purkinje cell synapses. These phenotypes may contribute to abnormal posture, balance, and motor learning of Math1-Cre;*Mea6*^F/F^ mice, as indicated in footprint, head inclination, balanced beam, and rotarod tests. These abnormal behaviors are not surprising, because cerebellar granule cell, which is the most numerous neuronal type in the brain, encodes massive sensorimotor information and is the common denominator of cerebellar information processing in normal motor behaviors and motor symptoms. It is reported that *Mea6* mutations are associated with Fahr’s syndrome (Lemos et al., 2011), which includes movement disorders (Oliveira et al., 2007). Therefore, the present work provides further insight into the roles of Mea6 in the development and function of the cerebellum and demonstrating Mea6 in granule cells may be more important to Fahr’s syndrome, because Math1-Cre;*Mea6*^F/F^ mice displayed severer movement phenotypes than Purkinje cell-targeted pCp2-Cre;*Mea6*^F/F^ mice (Wang et al., 2019).

It is shown that Mea6 is vital to the secretion of proteins including collagen, low-density lipoprotein (VLDL), and insulin (Saito et al., 2011; Saito et al., 2014; Wang et al., 2016; Fan et al., 2017). Further studies demonstrate that Mea6 regulates the sub-cellular transports of an exogenous component in cultured cortical neurons (Zhang et al., 2018) and endogenous proteins, including Slit2, BDNF, and semaphorin 3A, in Purkinje cells (Wang et al., 2019). The present work found that *Mea6* deletion in granule cells decreased the total and synaptic expressions of vGluT1 by detaining it in ER. Together, these studies suggest that Mea6 regulates the intracellular transport and maturation of proteins critical to neuronal development and function. In Purkinje cells, Mea6 regulates the expression of Slit2, BDNF, and semaphorin 3A, but not γ-pcdh, Robo2, and TrkB, all of which are critical to dendritic development (Wang et al., 2019). In granule cells, Mea6 regulates the expression of vGluT1 and BDNF, but not Rab3A, synapsin-1, Rim1, Munc18-1, synaptophysin, and Munc13-1, most of which are components of soluble N-ethyl-maleimide-sensitive fusion protein attachment protein receptor (SNARE) complex. These studies indicate a broad regulation capacity of Mea6 in neurons. Another point derived from the studies in Purkinje cells and granule cells is that Mea6 regulates part but not all proteins. From the study in Purkinje cells (Wang et al., 2019), we speculated that Mea6 influences secretary proteins but not membrane proteins. Similarly, Mea6 exhibits divergent roles in pre-synaptic proteins. However, the prevailing studies are not enough to answer a core question: What is the essence of divergent effects of Mea6 regulation of protein transport? Future study is needed to define the biological functions of Mea6 based on its structure and recognition capability.

Meningioma expressed antigen six ablation in Purkinje cells reduces Slit2 expression and thereby impairs the self-avoidance of Purkinje cell dendrites (Wang et al., 2019). Consequently, pCP2-Cre;*Mea6*^F/F^ mice display defective motor learning, while their gait is not changed (Wang et al., 2019). On contrast, Math1-Cre;*Mea6*^F/F^ mice exhibited more defects in posture, balance, and motor learning, indicating severer symptom in these mice. The difference between two types of mutants is an interesting question. Using biochemical, immunohistochemical, and electrophysiological assays, we demonstrated that Math1-Cre;*Mea6*^F/F^ mice suffered from a delayed migration of GCPs and impaired formation and function of parallel fiber-Purkinje cell synapses. It appears that dysfunctional granule cells induce more motor disorders rather than Purkinje cells. Nevertheless, this speculation might be paradoxical, because both Purkinje cells and granule cells are essential to the plasticity occurring at their innervations, which is a classic theory for cerebellum-related movements. A possible explanation for the phenotype difference is that self-avoidance defect only occurs at distal sites of parallel fibers in pCP2-Cre;*Mea6*^F/F^ mice (Wang et al., 2019) with the harm far milder than overall damage in Math1-Cre;*Mea6*^F/F^ mice. Alternatively, Mea6 may affect the functions of granule cells undiscovered in the present work, for example, the activation of mossy fiber-to-granule cell synapses, which is crucial to behavioral states as well.

In summary, our present findings provide further evidence to manifest the functions of Mea6 in cerebellar development and motor behaviors. We showed that *Mea6* deletion in granule cells impairs the transport of vGluT1 from ER to Golgi apparatus and the formation and function of parallel fiber-Purkinje cell synapses, which might underlie the damaged motor performance in Math1-Cre;*Mea6*^F/F^ mice. Last but not least, two caveats should be considered in the present work and need further studies: We had no clue to explain the cause of head inclination in Math1-Cre;*Mea6*^F/F^ mice, a novel observation with dysfunctional granule cells; We did not answer why Mea6 delayed but not impeded the migration of GCPs.

## Conclusion

The deletion of *Mea6* in cerebellar granule cells causes abnormal motor symptoms in footprint, head inclination, balanced beam, and rotarod tests. Math1-Cre;*Mea6*^F/F^ mice exhibited disrupted migration of GCPs and damaged parallel fiber-Purkinje cell synapses, which may be related to disrupted intracellular transport of vGluT1 and BDNF.

## Data Availability Statement

The original contributions presented in the study are included in the article/supplementary material, further inquiries can be directed to the corresponding author/s.

## Ethics Statement

The animal study was reviewed and approved by Animal Experimentation Ethics Committee of Zhejiang University.

## Author Contributions

X-TW and YS designed the research. X-TW, LZ, X-YC, and F-XX performed the research. Z-HX and X-YL provided the unpublished tools and techniques. X-TW, X-YC, and YS analyzed the data. X-TW, X-YL, and YS wrote the manuscript. All authors have read and approved the final manuscript.

## Conflict of Interest

The authors declare that the research was conducted in the absence of any commercial or financial relationships that could be construed as a potential conflict of interest.
